# A spark of genius and a flash of madness: Nikola Tesla and his struggles with mental illness

**DOI:** 10.1192/j.eurpsy.2024.664

**Published:** 2024-08-27

**Authors:** S. Jesus Magueta, A. L. Costa, G. Simões, A. I. Gomes, C. Madaíl Grego, P. Garrido

**Affiliations:** ^1^Departamento de Psiquiatria e Saúde Mental, Centro Hospitalar do Baixo Vouga, Aveiro, Portugal

## Abstract

**Introduction:**

An example of the unification of the contrast between artistic creativity and discipline of science, Nikola Tesla engineer and physicist, was also a prolific inventor that contributed to the transformation of modern society. Having resurfaced in the mainstream culture as a mythical figure, he appears to be enjoying a renaissance of posthumous recognition and praise. Throughaccounts available directly from his autobiography and descriptions offered by those who worked with him, the existence of the inventor´s *eccentricities* appear to reveal the existence of mental health disorder.

**Objectives:**

The authors explore Tesla and the psychopathology that accompanied him throughout his periods of brilliance and as well as hardship.

**Methods:**

The authors conducted a brief non-structured narrative literature review. The keywords used during the research, alone or in combination, included: Nikola Tesla, psychopathology and mental illness. The works consulted included: news articles, autobiographies and biographies. Of these, those that were written in the English language and deemed most pertinent to the explored theme were chosen for review in this work.

**Results:**

The popular image of the *mad scientist*, which describes a brilliant but solitary and eccentric individual focused on their work is one that could be applied to Tesla. Documents reveal that he suffered a nervous breakdown, as well as having symptoms that point to a probably presence of obsessive-compulsive disorder, of which included counting and cleanliness rituals, exacerbated by chronic insomnia.

**Conclusions:**

There appears to be anecdotal evidence pointing to an eventual relationship between creative genius and mental pathology. Although not formally evidenced through the scientific literature, exploring the life and accomplishments of Tesla serve as a significant example of a spark of genius perhaps ignited by mental illness. Tesla demonstrated suffering associated with his symptoms especially when considering the end of his life. At the time, adequate mental health interventions and treatments were not widely available, with his diagnosis probably being considered the quirks of genius and not the symptoms of disease.

**Disclosure of Interest:**

None Declared

